# Refractive errors and ocular findings in children and adolescents with mental disorders: a retrospective study

**DOI:** 10.1186/s12886-022-02704-4

**Published:** 2023-01-03

**Authors:** Liping Chen, Ling Sun, Caihong Xue, Shumao Li, Junjun Wang, Xia Shen, Shiyu Gao, Zixuan Zhou, Yuehe Xu, Shaocun Huang, Zhulin Li, Xiaoyan Yang, Yatu Guo, Wei Zhang

**Affiliations:** 1 Clinical College of Ophthalmology, Tianjin Medical University, Tianjin, China; 2 Tianjin Eye Hospital, Tianjin, China; 3 Nankai University affiliated Eye Hospital, Tianjin, China; 4 Tianjin Key Lab of Ophthalmology and Visual Science, Tianjin, China; 5 Tianjin Anding Hospital, Tianjin, China; 6 Mental Health Center of Tianjin Medical University, Tianjin, China

**Keywords:** Mental disorders, Refractive errors, Strabismus, Amblyopia, Children and adolesents

## Abstract

**Background:**

An increasing prevalence of mental disorders (MDs) has been reported among children and adolescents. However, only few studies have conducted ocular examinations, including those on refractive status, in these groups of patients. Thus, the purpose of this study was to evaluate the refractive status and ocular findings in children and adolescents with MDs compared with matched controls with similar socioeconomic backgrounds.

**Methods:**

A total of 178 participants with MDs and 200 controls were recruited between April 2021 and May 2022. All the children and adolescents underwent cycloplegic or noncycloplegic autorefraction and retinoscopy, slit-lamp biomicroscopy, and dilated fundus examinations. Ocular alignment was assessed using Hirschberg, Krimsky, or prism cover tests. The prevalence of refractive errors and ocular findings was the main outcome.

**Results:**

Twenty-seven percent of patients with MDs and 8% of controls had ocular findings, the most common of which were conjunctivitis, keratitis, and trichiasis. For refractive status, 70% (124/178) of patients with MDs had myopia ≤-1.00 DS, and 2% (4/178) had hyperopia ≥+2.00 DS. In the control group, 70% (140/200) of patients had myopia ≤-1.00 DS, and 1% (2/200) had hyperopia ≥+2.00 DS. No differences were observed between the MD and control groups. However, the patients in the MD group (14.25±2.69 years) were significantly more susceptible to strabismus (*P*<0.05) and amblyopia (*P*<0.01) than those in the control group (13.65±3.04 years). There was a substantial difference between the two groups in the time spent on screen-based devices (*P*<0.001). Furthermore, mental retardation (OR=3.286, *P*<0.01), emotional disorders (OR=2.003, *P*<0.01), and adjustment disorders (OR=2.629, *P*<0.01) were associated with an increased risk of amblyopia. Depression (OR =1.362, *P*<0.01) and emotional disorders (OR=2.205, *P*<0.01) were associated with a higher prevalence of strabismus.

**Conclusion:**

Ophthalmological examinations should be performed in children and adolescents with MDs because MDs are associated with a high prevalence of refractive errors and ocular diseases. Detection and intervention of ocular and refractive findings in children and adolescents with MDs are necessary and effective in alleviating the economic burden in healthcare and improving individuals' quality of life

## Background

Mental disorders (MDs) in childhood refer to anxiety disorder, depression, schizophrenia, bipolar and related disorders, mental retardation, and substance-related and addictive disorders, with onset at ages below 18 years [1]. Some MDs are specific to children and adolescents, such as pervasive developmental disorders, attention deficit hyperactivity disorder (ADHD), conduct disorders, and childhood emotional disorders. According to data from the World Health Organization in 2005, up to 20% of adolescents worldwide suffer from MDs, and 50% of adult MDs start in adolescence [2].

Refractive errors and visual impairments in adults with learning disabilities have been extensively studied [3–5]. Wang et al. performed vision screening on 168 Chinese children with autism spectrum disorders (ASD), aged between 3 and 8 years. They found that children with ASD had a higher incidence of strabismus (16.1%) [6]. Su demonstrated that adolescents with amblyopia had a greater risk of ADHD [7]. However, only few studies have focused on refractive status and ophthalmologic abnormalities in such populations.

In this study, we investigated 178 patients aged 6–18 years admitted to the adolescent psychology outpatient department in Tianjin Anding Hospital. This study aimed to assess the refractive status and ocular findings in children and adolescents and with MDs compared to matched controls with similar socioeconomic backgrounds.

## Materials and Methods

The study strictly adhered to the Declaration of Helsinki and was approved by the Ethics Review Committees of Tianjin Anding Hospital and Tianjin Eye Hospital, China. Written informed consent was obtained from the parents of all participants at the beginning of the study, and mental health professionals enrolled the participants at a mental health institution in Tianjin. A senior psychiatrist diagnosed the MDs. We evaluated mental illnesses, including anxiety disorder, depressive disorder, emotional disorder, mental retardation, bipolar disorder, ASD, ADHD, and schizophrenia. One hundred seventy-eight patients with MDs aged 6–18 years old (86 boys, 92 girls; 14.25±2.69 years [mean±SD]) who were admitted to the children and adolescents psychology department of Tianjin Mental Health Center from April 2021 to May 2022 were included. Ocular examinations and psychological procedures were assessed. All patients underwent a complete ophthalmologic examination, including visual acuity, best-corrected visual acuity, cycloplegic or noncycloplegic refraction, slit-lamp examination of the anterior segment, fundus examination, dominant eye fixation, ocular motility (monocular and binocular eye movement), and the Titmus stereoacuity tests. Refractive status was determined based on cycloplegia (1% cyclopentolate eye drops were used in patients under 15 years of age). The spherical equivalent (SE) of the refractive errors was calculated using the following formula: spherical refraction + 0.5 × cylindrical refraction [8]. The vector presentation of astigmatism was presented as J0 and J45 and calculated according to the following formula: J0 = (−C/2) × cos(2×θ); J45 = (−C/2) × sin(2×θ )[8]. Amblyopia is a unilateral or, less often, bilateral reduction of best-corrected visual acuity that usually occurs in the setting of an otherwise normal eye [9]. We defined unilateral amblyopia as a 2-line or more difference with the best-corrected interocular VA. Refractive errors is classified with myopia, hyperopia, astigmatism and anisometropia. The age and visual demands of the individual patient also be taken into account.

Horizontal and vertical deviations were measured using prism and alternate cover tests, respectively, and the time spent sleeping and using screen-based devices was recorded. The age- and sex-matched control group comprised 200 participants (95 boys, 105 girls; 13.65± 3.05 years [mean±SD], age range 6–18 years), with sex distribution (χ^2^ =0.01, *P* = 0.919) and mean ages (14.25±2.69, 13.65±3.04, *P*>0.05) being similar among the two groups, without any mental illness or psychiatric problems.

### Statistical analysis

SPSS version 25.0 software (SPSS Inc., Chicago, IL) was used for statistical analysis. The measurement variables are presented as mean ± SD, while the quartiles were used to present categorical data. If variables were normally distributed, the Student’s t-test was used to compare them; otherwise, the Mann–Whitney U test was used. In addition, χ^2^ tests were used to compare categorical data. The generalized estimating equation model and linear regression were used to compare the SE, J0, J45, and diopter cylinder of the two eyes between the MD and control groups. Multiple logistic regression was performed to determine the factors (anxiety disorder, depressive disorder, emotional disorder, mental retardation, bipolar disorder, ASD, ADHD, and schizophrenia ); confounders included sex, age, parental education level, sleeping time, and time spent watching television) associated with the occurrence of MDs.

## Results

### Refractive errors

**Fig. 1:**
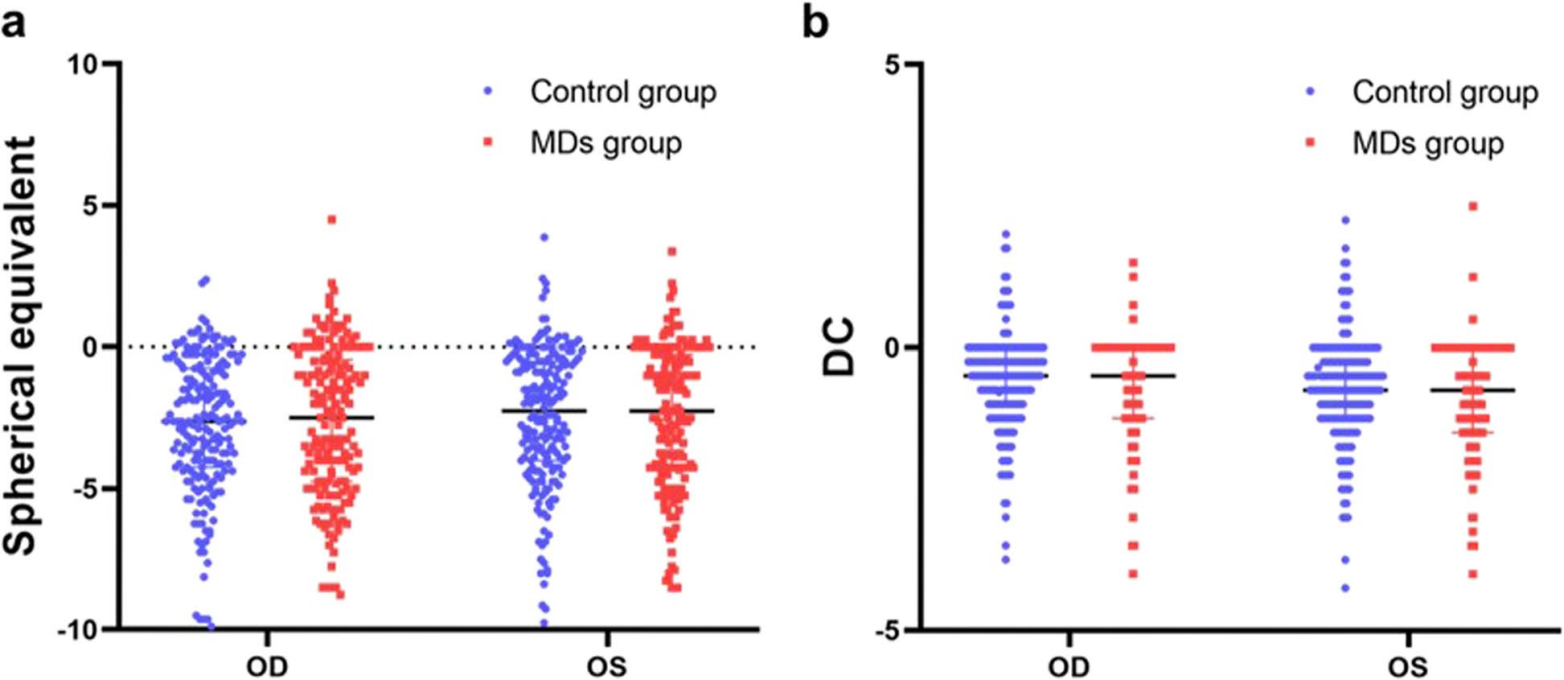
Comparison of SE (**a**) and DC (**b**) of OD and OS (DC:diopter cyclinder)

**Fig. 2:**
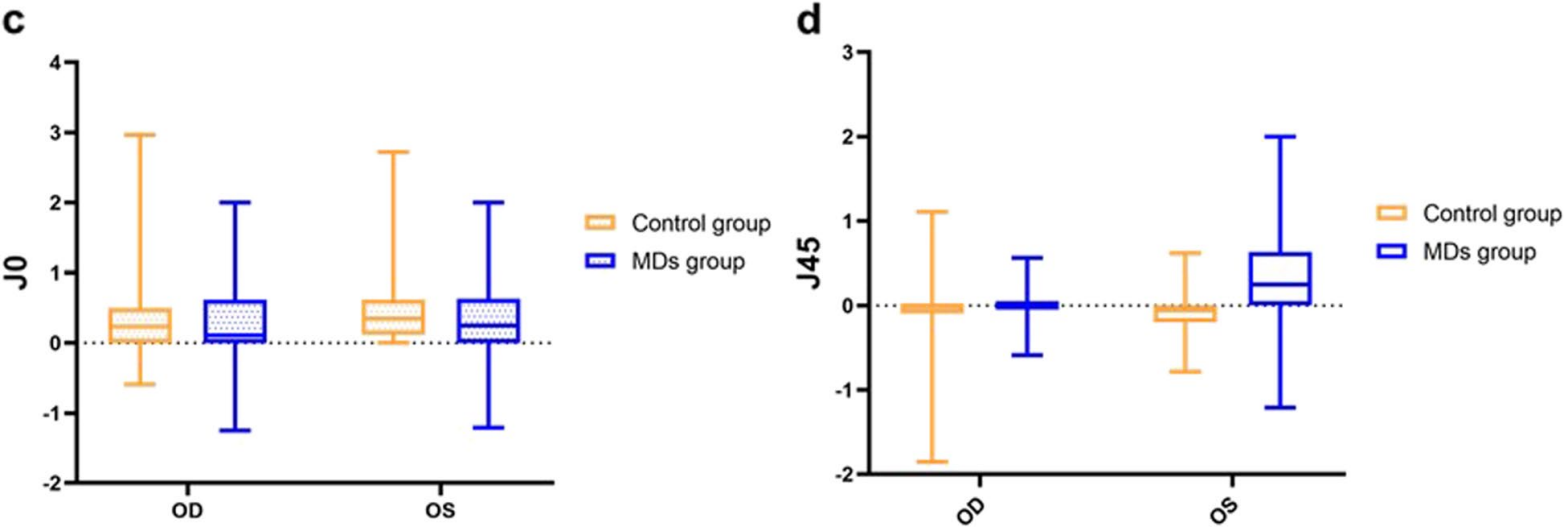
Comparison of J0 (**c**) (**d**) of OD and OS

Cycloplegic refraction was measured in 216 eyes of 108 adolescents aged < 15 years. Non-cycloplegic refraction was conducted in 140 eyes of 70 adolescents. In the MD group, the SE of the right eye was -2.64±2.61 and that of the left eye was -2.47±2.54. There were 49 cases of anisometropia and 12 cases of amblyopia in the MD group (Table 1). The time spent sleeping and using screen-based devices (television, smartphones, and computers) is presented in Table 2. In the control group, cycloplegic refraction was successfully measured in 232 eyes of 116 adolescents aged < 15 years. Noncycloplegic refraction was performed in 84 adolescents. The SE of the right eye was -2.79±2.37 and that of the left eye was -2.47±2.37. There were 65 cases of anisometropia and two cases of amblyopia in the control group (Table 1). The amblyopia risk in children with MDs was significantly higher than that in the control children (*P*< 0.01). High myopia ≥ -6.00 DS or astigmatism ≥ 2.50 DC was diagnosed in 20 cases and 18 cases both in the MD and control groups, respectively. There were no statistically significant difference in the SE  and DC between the two groups (*P*>0.05, Fig. 1) Anisometropia was defined as the difference in refractive error between eyes exceeding 1.0 diopter (D) or cylinders more than 1.5 D. The anisometropia types were classified as spherical, astigmatic, and mixed. In this study, there was no statistically significant difference in J0 between the two groups, whereas the difference between the groups for J45 was statistically significant (*P*<0.01) (Fig. 2). J0 represents the astigmatism component in the horizontal or vertical direction, and J45 represents astigmatism at 45^°^ and 135^°^.

**Table 1:** Age and sex distribution and opthalmic findings in MDs and controls

	MDs group (*n*=178)	Control group (*n*=200)	
Age (years)	14.25±2.69	13.65±3.04	*P* >0.05&
Sex			
Male	86	95	*P* >0.05*
Female	92	105	
SE (DS)			
OD	-2.64±2.61	-2.79±2.37	*P*=0.779#
OS	-2.47±2.54	-2.47±2.37	
J0(D)			
OD	0.63±1.94	0.15±0.09	*P*=0.11#
OS	0.01±0.17	0.31±0.09	
J45(D)			
OD	0.39±2.27	-0.02±0.16	*P*<0.01#
OS	-0.15±0.21	0.23±0.39	
DC			
OD	-0.68±0.90	-0.59±0.12	*P*=0.25#
OS	-0.84±0.93	-0.73±1.04	
Anisometropia			
Spherical	44	63	*P* >0.05*
Astigmatic	2	0	
Mixed	3	2	
None			
Amblyopia			
(+)	12	2	0.005*
(-)	166	198	
Corrected VA	logMAR0.03±0.06	log MAR 0.02±0.03	*P* >0.05†
Strabismus			
Horizontal	10	4	0.011*
Vertical	4	1	
Nystagmus	1	0	
Stereoacuity (seconds)	70(70, 140)	40(40, 50)	*P*<0.01†

* chi-square test;† Mann–Whitney U test; & T tests; #, GEE; *SE* spherical equivalent, *VA* visual acuity

**Table 2:** Time spent on sleeping and screen-based devices with MDs and controls

Characteristic	MDs group(n=178)	Control group(n=200)	*P*
Time spent on sleeping			
<9 hrs/day	82	79	0.165*
≥9 hrs/day	96	121	
Time spent on screen-based devices			
<2 hrs/day	75	188	*P*<0.001*
≥2 hrs/day	103	22	

* chi-square test

### Strabismus

Fourteen children and adolescents with strabismus were included in the MD group. The most common type of strabismus was intermittent exotropia (IXT), which was diagnosed along with an emotional disorder in 9 cases. The horizontal deviation ranged from 18 PD to 50 PD (-30.11±2.26 PD at 33 cm, and 28.44±2.36 PD at 6 m). Four patients of exotropia combined with hypertropia, abnormal head posture, and ocular motility showed overaction of the inferior oblique. The horizontal deviation was -21.75±7.14 PD at 33 cm and 18.00±2.31 PD at 6 m, and the vertical deviation was 10.50±4.43PD at 33 cm and 10.00±4.90 PD at 6 m. Additionally, nystagmus occurred in one adolescent (1/14) in the MD group. In the control group, four children and adolescents presented with intermittent exotropia and one with hyperopia. Significant statistical differences in strabismus were shown between the two groups (*P*=0.011).

### Stereoacuity

In the MD group, the Titmus stereo test of fewer than 100 seconds of arc was found in 129 cases, while 46 patients showed a stereo test from 400 to 100 seconds of arc. In the control group, the stereoacuity in 188 of 200 cases was less than 100 seconds of arc. Stereoacuity analysis revealed significant differences between the MDs and control groups (*P*<0.01).

### Ocular disorders

Ocular disorders were detected in many adolescents in the MDs and control groups. Conjunctivitis was the most common condition in the two groups (24% in the MD group and 5% in the control group), followed by keratitis and trichiasis. The findings of each ocular disorder are presented in Table 3. The results of the multiple logistic regression analysis of risk factors for significant refractive error, amblyopia, and strabismus are presented in Table 4. Among 178 MD patients in our study, mental retardation was associated with an increased risk of amblyopia (OR=3.286, *P*<0.01). There was an increased risk of amblyopia in emotional (OR=2.003, *P*<0.01) and adjustment disorders (OR=2.629, *P*<0.01). Depression (OR =1.362, *P*<0.01) and emotional disorders (OR=2.205, *P*<0.01) were also associated with an increased risk of strabismus.

**Table 3:** Ocular disorders with MDs and control group

	MDs group(n=178) n%	Control group(n=200) n%
Conjunctivitis	24(13%)	10(5%)
Keratitis	12(7%)	3(2%)
Optic disc edema	1(0.5%)	0
Corneal endothelial dystrophy	1(0.5%)	0
Trichiasis	7(4%)	3(2%)
IOL implantation	2(0.5%)	0

*IOL *intraocular lens

**Table 4:** Odds ratios for risk factors for refractive error, amblyopia, and strabismus in adolescent with mental disorder by multiple logistic regression

Risk factor	Odds ratios (95% confidence interval)		
	Significant refractive error	Amblyopia	Strabismus
Depression	3.617 (0.869, 15.06)	1.885 (1.583, 49.857) *P*=0.013	1.362 (2.256, 24.019) *P*=0.001
Mental retardation	N/A	3.286 (3.974, 136.456) *P*=0.001	N/A
Emotional disorders	2.091 (0.995, 4.395)	2.003 (1.792, 20.108) *P*=0.004	2.205 (3.386, 11.372) *P*=0.001
Adjustment disorder	4.415 (0.958, 20.348)	2.629 (3.223, 107.687 *P*=0.001	N/A

## Discussion

A previous study has shown that 14.5% of adolescents aged 7–17 years meet the criteria for at least one MD related to mental impairment, and the comorbidity rate of MDs in children and adolescents is higher than that in adults [10]. Another study showed that anxiety disorders are the most common MDs in adolescents, followed by behavioral, affective, and substance use disorders [11]. However, our study showed that emotional disorders were the most common (46%), followed by anxiety and depression.

MDs or mental health problems during adolescence increase the risk of vision problems in early adulthood. The present study analyzed the spherical diopters, cylindrical diopters, and the prevalence of anisometropia in adolescents with MDs as well as a control group. Refraction is generally performed following cycloplegia or noncycloplegia. For children and adolescents under the age of 15 years, cyclopentolate hydrochloride (1%) was used to evaluate the refractive status and corrected visual acuity. We found that the MD group was significantly more susceptible to astigmatism than the control group. Nielsen and Das reported a higher prevalence of hypermetropia and astigmatism in adolescents with a developmental delay than in normal adolescents [12, 13]. Chang et al. reported that the prevalence of high myopia was 4.1% and high astigmatism was 19.8% among Taiwanese students with cognitive impairment aged 15–23 years [7]. However, we found that the prevalence of high myopia was 11.2% in the MD group, similar to that in the control group (10%). The prevalence of myopia in the MD group was not higher than that in the control group. Moreover, the age range was 6–18 years old, covering the age at which myopia is expected to occur. Yan et al. found that a decline in near stereoacuity can result in significant anxiety and depression [14]. Visual impairment and loss of binocular function are related to symptoms of anxiety and depression [15]. The stereoacuity results in our study largely agreed with their views.

There is an association between internet addiction (IA), anxiety disorders, and ADHD, males were associated with IA, whereas there is no significant correlation between age and IA [16]. Studies have shown that depression may be a risk factor for IA [14, 17, 18]. In our study, the time spent on screen-based devices significantly differed between the MD and control groups (*P*<0.001). We hypothesized that IA might contribute to an increase in the rate of myopia. Further studies should investigate the association between IA and myopia in the MD group.

Friedman et al. reported that the prevalence of strabismus is 1.2–6.8% in children aged 6–17 years in a large sample of preschool children [14] .Other studies have reported that the prevalence of strabismus ranges from 2.1% to 3.6% [15, 19]. These studies also found a general prevalence of amblyopia ranging from 0.8% to 2.6% and a significantly greater prevalence in Hispanic adolescents. Ophthalmic disorders were found in 71% of adolescents with ASD, strabismus (32%), amblyopia (19%), and significant refractive errors (42%) [20]. Lin et al. reported that adolescents aged 6–17 years with strabismus have a higher prevalence of depression, anxiety, and alcohol consumption in China [12]. Several studies have suggested that patients with strabismus may be at a higher risk of developing mental illness [14, 21, 22]. A study conducted in Germany found that children with strabismus had more severe mental health problems [11]. Another cross-sectional study concluded that there is a moderate association between strabismus and MDs [23]. Esotropia, exotropia, and hypertropia increase the odds of mental health issues. Similarly, the results of multivariate logistic regression models indicated that mental retardation, emotional disorders, and adjustment disorders were associated with increased amblyopia risk. The data in this study show that depression and emotional disorders are also associated with an increased risk of strabismus. Adolescents with strabismus are more likely to worry about their eyes, have negative attitudes and misconceptions, and endure increased psychological distress.

Whether having exotropia or esotropia is more likely to cause a child to develop MDs is controversial. In the US, intermittent exotropia (2.7 times) is more likely to be diagnosed with a mental health issue in young adulthood [24] .Mohney investigated the prevalence and types of psychiatric disorders diagnosed in early adulthood among adolescents with strabismus, patients with exotropia were 3.1 times more likely to develop mental illness by early adulthood than those in the control group [22]. Exotropia is more likely to cause a greater number of MDs than esotropia. In contrast, Olson demonstrated that adolescents with congenital esotropia have a 2.6 times greater chance of developing mental illness [21]. Wang implied that adolescents with ASD tend to have more esodeviation (66.7%) than exodeviation (20.2%) [6]. Our results suggest that adolescents with MDs are more likely to have strabismus with exotropia, similar to the prevalence of strabismus in China. Studies on the psychosocial impact of strabismus have reported no differences between subjects with exotropia and those with esotropia [25, 26]. A larger number of samples should be included in further investigations.

## Conclusion

In summary, it is worthwhile to perform ophthalmological examinations in adolescents with MDs since MDs are related to a high prevalence of refractive errors and ocular diseases. Detection and intervention of ocular and refractive findings in children and adolescents with MDs are necessary and effective in alleviating the economic burden in healthcare and improving individuals' quality of life. Further, there should be increased awareness among psychiatrists about the surge in refractive errors and ophthalmologic abnormalities in children with MDs.

## Data Availability

All data analyzed during this study are included in this published article.

## Abbreviations

MDs: mental disorders
ADHD: attention deficit hyperactivity disorder
ASD: autism spectrum disorders
SE: spherical equivalent
IXT: intermittent exotropia
IA: internet addiction
DS: diopter sphere
DC: diopter cylinder
